# Balance beam crossing times are slower after noise exposure in rats

**DOI:** 10.3389/fnint.2023.1196477

**Published:** 2023-07-11

**Authors:** Dylan Bartikofsky, Mikayla Jade Hertz, David S. Bauer, Richard Altschuler, W. Michael King, Courtney Elaine Stewart

**Affiliations:** ^1^Lieutenant Colonel Charles S. Kettles VA Medical Center, Ann Arbor, MI, United States; ^2^Department of Otolaryngology/Head-Neck Surgery, Kresge Hearing Research Institute, University of Michigan, Ann Arbor, MI, United States

**Keywords:** vestibular, noise exposure, noise-induced vestibular loss, balance, mobility, motor impairment

## Abstract

**Introduction:**

The vestibular system integrates signals related to vision, head position, gravity, motion, and body position to provide stability during motion through the environment. Disruption in any of these systems can reduce agility and lead to changes in ability to safely navigate one’s environment. Causes of vestibular decline are diverse; however, excessive noise exposure can lead to otolith organ dysfunction. Specifically, 120 decibel (dB) sound pressure level (SPL) 1.5 kHz-centered 3-octave band noise (1.5 kHz 3OBN) causes peripheral vestibular dysfunction in rats, measured by vestibular short-latency evoked potential (VsEP) and reduced calretinin-immunolabeling of calyx-only afferent terminals in the striolar region of the saccule. The present study examined the functional impact of this noise exposure condition, examining changes in motor performance after noise exposure with a balance beam crossing task.

**Methods:**

Balance beam crossing time in rats was assessed for 19 weeks before and 5 weeks after noise exposure. Balance beam crossings were scored to assess proficiency in the task. When animals were proficient, they received a single exposure to 120 dB SPL 3-octave band noise.

**Results:**

During the initial training phase slower crossing times and higher scores, including multiple failures were observed. This was followed by a period of significant improvement leading to proficiency, characterized by fast and stable crossing times and consistently low scores. After noise exposure, crossing times were significantly elevated from baseline for 4-weeks. A total of 5 weeks after noise exposure, crossing times improved, and though still trending higher than baseline, they were no longer significantly different from baseline.

**Discussion:**

These findings show that the noise-induced peripheral vestibular changes we previously observed at cellular and electro-physiological levels also have an impact at a functional level. It has been previously shown that imbalance is associated with slower walking speed in older adults and aged rats. These findings in noise-exposed rats may have implications for people who experience noisy environments and for seniors with a history of noise exposure who also experience balance disorders and may be at increased fall risk.

## Introduction

We previously demonstrated reduced amplitude and prolonged latency of vestibular short-latency evoked potential (VsEP) responses following a single 6-h exposure to 120 dB sound pressure level (SPL) 3-octave band noise (3OBN) centered at 1.5 kHz in rats. Furthermore, after noise exposure, calyx-only afferent terminal labeling with calretinin was reduced in the saccule, but calyces were otherwise unaffected 1-month after noise exposure. Hair cells were also unaffected 1-month after noise exposure, suggesting that the noise exposure paradigm preferentially impacted immunolabeling of calyx only afferent terminals, sparing afferent terminal structures and hair cells (Stewart et al., 2018, 2020). This finding is of particular importance for understanding how noise produces immediate dysfunction in the vestibular periphery, but the behavioral consequences of peripheral dysfunction are unclear. Thus, a primary goal of this work is to understand if there are short-term and/or long-term effects of noise exposure on balance, agility, and overall motor performance following noise exposure that has been previously demonstrated to impact the vestibular periphery. Specifically, this work will investigate the functional consequences of noise exposure on balance beam crossing performance in rats.

Balance beam crossing experiments have been used to study motor performance in a variety of rodent models, including vestibular-specific deficits associated with noise exposure in mice (Tamura et al., 2012). Tamura et al. (2012) demonstrate that a 1-month exposure to 70 dB SPL 100 Hz noise caused changes in gait, slower balance beam crossing times, vestibular hair cell loss, and elevated markers of oxidative stress. This study did not utilize training and did not track recovery. Although this was adequate for the goals of the Tamura et al. (2012) study, longitudinal studies of aging or extended evaluation of models that investigate the time course of damage and recovery require stable and proficient balance beam crossing behavior over an extended time period, in some cases, weeks, months, or even years. Importantly, this study evaluates the effect of prolonged training on crossing proficiency and crossing time. Although many previous studies document limited training, this work highlights the importance of establishing balance beam crossing proficiency prior to experimental manipulations.

Balance beam tasks are sensitive to very subtle signs of balance and motor impairment that tests such as rotarod may miss (Luong et al., 2011). This test has successfully identified motor impairment in rodents with cortical-cholinergic and striatal-dopaminergic lesions (Kucinski et al., 2013), chemical exposure (Negishi-Oshino et al., 2019; Sen et al., 2020), traumatic brain injury (Lyeth et al., 1993), exposure to magnetic fields that impact hippocampal and vestibular function (Tkac et al., 2021), cerebellar abnormalities (Koirala and Corfas, 2010), unilateral vestibular deafferentation (Heskin-Sweezie et al., 2010), central vestibular lesions (Modianos and Pfaff, 1976), genetic mutations that affect the vestibular system (Hanada et al., 2018; Takimoto et al., 2018; Manes et al., 2019; Ono et al., 2020), and in rodent aging models (Wallace et al., 1980; Drucker-Colín and García-Hernández, 1991; Tung et al., 2016).

Ono et al. (2020) demonstrate a particularly interesting phenomenon with relevance to our previous work. In mice that are unable to produce Cyp26b1, which is associated with degradation of retinoic acid, striolar/central zones in vestibular end organs are compromised and mice walk more slowly across a balance beam (Ono et al., 2020). This study highlights the value of the balance beam crossing task in assessment of vestibular-mediated motor performance in genetic models that produce complete lesions of the otolith organ striolar zones and canal cristae central zones. We anticipated less profound effects on balance beam crossing following noise exposure since the noise-induced changes appear to be specific to calyx-only afferent terminals in the otolith organs. We analyzed balance beam crossing times with a scoring system that accounted for stops during crossings to evaluate training level and noise-induced damage. The scoring system was a useful tool to determine if the stopping behavior caused crossing to occur more slowly or if crossing time was slower because rats moved more slowly regardless of stops.

## Materials and methods

Our preliminary baseline studies on balance beam crossing in Long-Evans rats found that female rats reached training proficiency more rapidly than males and had less variability after 12 weeks of training. This may be due in part to their smaller size and slower growth during the training and post-noise period. Therefore, the current study used only females. Twenty female Long-Evans rats weighing 262 ± 12 g and 11.5 ± 0.8 weeks old (Charles River Laboratories) at the start of the experiment were pair housed on a 12:12-h light-dark cycle (lights on at 0600 and off at 1800) with *ad libitum* access to food and water. All procedures were carried out in accordance with National Institutes of Health guidelines and were approved by the Institutional Animal Care and Use Committee at the LTC Charles S. Kettles VAMC in Ann Arbor, MI, USA.

**FIGURE 1:**
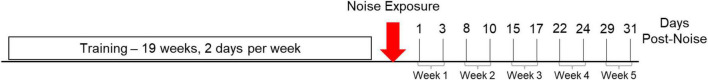
Experimental timeline. Rats are trained twice per week over a 19-week period. At the end of training, rats are noise exposed (red arrow) and balance beam crossing resumes on the following day (1 day post-noise). After noise exposure, 10 trials per day continue to be collected twice per week for 5 weeks or 31 days.

Rats were trained to cross a balance beam until crossing times stabilized under 1.5 s and did not vary significantly from the previous week of data. Crossing scores that took into account stopping behavior were also used to track training progress and proficiency. Each run, or trial, was scored on a 3-point basis: 1 = no stops, 2 = 1–3 stops, and 3 = 3 + stops or a crossing time of >10 s resulting in failure. Each rat completed this task 10 times, twice per week. Ten trials for each of 20 rats for two experimental days were collected each week (240 noise rat and 160 control rat trials per week) and then averaged and compared with repeated measures ANOVA. A mean score of 1.2 for an individual rat was considered the cut-off for proficiency, and most rats fell between mean scores of 1.0–1.2, where a score of 1.0 is a perfect day, and higher scores (up to a mean of 3.0, or a score of 3 for every trial) indicated a need for additional baseline training. Crossing proficiency was defined as an average score of <1.2 across all training baselines. The week of baseline data collected immediately prior to noise exposure was used for analysis of crossing time and scores but 19 weeks of baseline data were collected to assess training progress over time. Because no treatment had occurred, control and noise rats’ baseline timepoint were pooled and compared with repeated measures ANOVA and the weekly baseline timepoint were analyzed with multiple pairwise comparisons. One day after noise exposure, and for 5 weeks after noise exposure, rats continued to perform 10 balance beam crosses two times per week (for timeline, see Figure 1). All the data collected for each week was pooled as one experimental timepoint for each group (Control and Noise). Data collected 1 week prior to noise exposure and each post-noise timepoint starting 1 day after noise exposure were compared with repeated measures ANOVA followed by multiple pairwise comparisons to explore differences at each timepoint between control and noise rats and within each treatment group vs. their own baselines.

### Balance beam design

**FIGURE 2:**
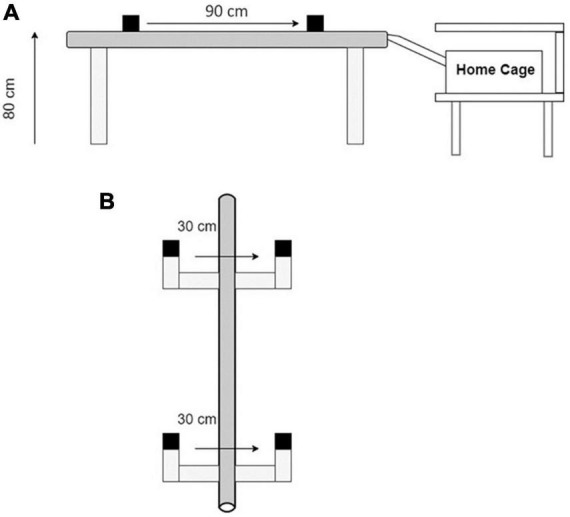
The balance beam apparatus. **(A)** A 90 cm high conduit with 2 IR sensor assemblies spaced 90 cm apart. **(B)** A top-down view of the start and stop sensors (S1 and S2). Each sensor assembly has an infrared beam spaced 30 cm from a sensor. Rats are placed 55 cm behind S1 at the start of the task. Each trial is initiated when the rat walks through S1 disrupting the light-sensor connection. Each trial is terminated when the rat walks through S2 or the rat refuses to cross for more than 10 s after walking through the S1 sensor.

The balance beam consisted of an 80 cm high conduit (2.0 m, 4.2 cm outer diameter, Figure 2A) with 2 IR sensors (30 cm from light to sensor, Figure 2B) indicating the start position (S1) and stop position (S2) sensors. The beam was supported by 4 bases. A polylactic acid (PLA) bridge connected the balance beam to the rats’ home cage, placed under a shelf to provide shelter as reinforcement (Figure 2A). This familiar and sheltered environment provided sufficient reinforcement to obtain consistent trials after the training period and motivated rats to complete the task. Fruity gems (Bio-Serv, Flemington, NJ, USA) were provided as an additional reinforcer. The sensors are spaced 90 cm apart and this length was used to score and time each crossing (Figure 2A). The rats were placed 55 cm behind the S1 sensor; the time began once the rat crossed S1 and finished when the rat crossed S2 (Figure 2). Therefore, the total beam length traversed per trial was 145 cm. Voltage changes were measured at each sensor using a DATAQ DI-2108 acquisition unit (Dataq Instruments Inc., Akron, OH, USA). A channel for each voltage collected the data for the entire duration of each experimental day for each rat. Files for each rat were converted to a .csv file in bins of (10 ms) and imported into MATLAB for analysis. Spikes in voltage were measured at their peaks to determine start and stop times for each of the two sensor channels. The time for each of the 10 start and stop peaks was recorded and stored for analysis.

### Noise exposure calibration

Sound level was measured with a calibration microphone kit (PCB 378C01, PCB Piezotronics, Depew, NY, USA), connected to a 1-channel battery-powered ICP^®^ sensor signal conditioner (480C02, PCB Piezotronics, Depew, NY, USA). The signal conditioner sent output to an oscilloscope (1054z, Rigol, Portland, OR, USA). The oscilloscope was used to collect sound level measurements around the expected frequency range of the noise exposure. Using the fast Fourier transform (FFT) function with a Blackman filter, a spectrum was generated. Measurements were taken from the generated spectrum every 100 Hz from 0 to 5.5 kHz using horizontal and vertical cursors in the viewing window. Measurements were manually recorded and plotted in MATLAB to give a noise spectrum with a peak intensity of approximately 120 dB SPL and a frequency range of approximately 0.5–4 kHz, as described previously (Stewart et al., 2020).

### Noise exposure

The 120 dB SPL noise used in this experiment is at a level similar to a siren, sporting event, or concert. It Is not unusual to encounter this sound level; however, sounds that reach this level are unsafe without hearing protection for any amount of time and may produce immediate auditory and vestibular injury. A total of 1.5 kHz 3-octave band noise activates the upper 20% of the rat cochlea and lower hearing frequency range. It is known that lower frequencies within this range activate the vestibular periphery (Curthoys et al., 2018). The duration selected has been previously demonstrated to produce a permanent reduction in VsEP responses and calretinin immunolabeling calyx-only afferent terminals (Stewart et al., 2020), but is unlikely to be encountered as a single “dose” in everyday life. Awake and freely moving rats were placed in a multi-level rat habitat with water and chow provided *ad libitum*. The rat habitat was placed in a modified ETS-Lindgren RE-141 booth with upgraded 11-gauge inner skins for increased external noise reduction and internal reflection (ETS-Lindgren, Cedar Park, TX, USA). The booth was illuminated with an overhead light throughout the 6-h noise exposure period, and a monitoring window was used to monitor overall condition of rats being exposed to noise at least every hour. Overall, rats seem to tolerate noise exposure well, and we observe normal grooming, eating, drinking, sleeping, and climbing behaviors during the noise exposure period. To reach 120 dB SPL noise level, we used an attenuator knob to elevate noise level from ∼80 to 120 dB SPL over approximately 5 min. Noise was generated from an audio clip developed in Adobe Audition and converted to a .smr file that could be output in Spike2 via a CED Micro1401 data acquisition unit (Cambridge Electronic Design, Cambridge, England). A DAC port on the Micro1401 sent the signal to a Saga Pro stereo power amplifier (800W @ 8 Ohms) and the power amplifier drove a LaVoce WAF 123.03 12″ Ferrite 8 Ohm Professional Woofer (LaVoce Italiana, Zhejiang, China). The woofer delivered 1.5 kHz centered 3-octave band noise (3OBN) at a peak of around 120 dB SPL over a frequency range of approximately 600–4200 Hz for 6 h. At the end of the noise exposure, rats were carefully inspected then returned to their home cage and taken to the colony room to recover overnight. Static vestibular signs including circling, head tilt, unprovoked nystagmus, and lowered body posture were not observed at any time before or after noise exposure. Control rats did not receive noise exposure.

## Results

### Effect of extended training

**FIGURE 3:**
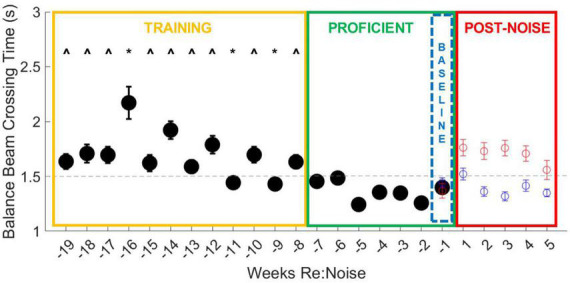
Balance beam crossing times improve with training. During the initial 12-week training period (yellow box), all weeks are different (^) from more than one proficient timepoint (green box). Weeks −16, −11, and −9 are also significantly different from other training timepoint (*). Week −16 is significantly slower than most other timepoint. This may be because there were 27 trials that took longer than 3 s, including six failures due to crossing times that exceeded 10 s. In weeks −11 and −9, crossing times were significantly faster and fall below the cut-off time for proficiency, suggesting improvement in training. Taken together, these data show that extended training does produce a measurable improvement in crossing time and underscores the importance of training prior to experimental treatments. The final week of baseline data (blue dashed box) was used as the baseline for crossing times for 5 weeks after noise exposure (Figure 5). The data from Figure 5 are also plotted for comparison with the training data. Although control rats continue to show stable and proficient balance beam crossing times, noise exposed rats’ crossing times become slower, reaching mean crossing times similar to training timepoint.

**FIGURE 4:**
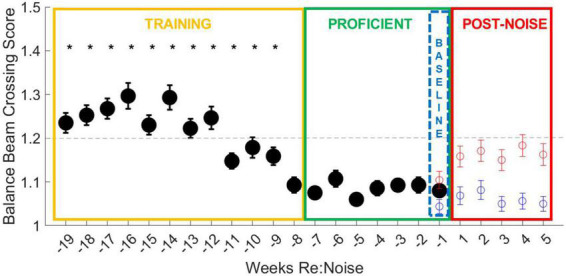
Balance beam crossing scores improve with training. During the initial 12-week training period (yellow box), all weeks except for week −8 are significantly different from more than one other training and more than one proficient timepoint (*). Crossing scores are less variable, even in early training than crossing times, and are more consistent from week to week. By the end of the training period (weeks −11 to −8), crossing scores are more consistent and lower, falling below the cut-off for proficiency, suggesting improvement in training. The final week of baseline data (blue dashed box) was used as the baseline for crossing scores for 5 weeks after noise exposure (Figure 6). The data from Figure 6 are also plotted for comparison with the training data. Although control rats balance beam crossing scores remain lower than noise exposed rats during the post-treatment timepoint, noise exposed rats’ mean balance beam crossing scores do not exceed the cut-off for proficiency. This may suggest that crossing scores are a less sensitive measure of noise-induced motor impairment than balance beam crossing times.

There is a training period (yellow box, Figures 3, 4), a proficient period determined by crossing score and stable reduction in crossing times (green box, Figures 3, 4), and the baseline week of data used for analysis of post-noise changes (blue dashed box, Figures 3, 4) which shows a small and not significant increase in crossing time with a stable score. Balance beam crossing training lasted for 19 weeks. Rats initially had variable and slower crossing times that varied significantly from proficient timepoint. Week −16 was significantly slower than training and proficient timepoint due to a few rats’ elevated crossing times. Near the end of the training phase and before crossing times stabilized, weeks −11 and −9 were significantly faster than many training timepoint, but also significantly different from some proficient timepoint (Figure 3).

Similar to crossing times, crossing scores were initially significantly higher than later training timepoint; however, crossing scores improved more rapidly than crossing times (Figure 4). As crossing scores decreased at the end of the training period and rats reached increasingly stable proficiency scores, crossing times also stabilized under 1.5 s. Baseline and post-noise timepoint are individually plotted for control (blue circles) and noise exposed groups (red circles) for comparison. Noise exposed rats’ crossing times return to speeds similar to the training phase and above the cut-off time for proficiency. This is not the case for crossing scores. Although the crossing scores are elevated from baseline, means do not exceed the cut-off score of 1.2 for training proficiency. This suggests that crossing score may be a useful tool for tracking training, but once crossing scores stabilize, noise exposure appears to have less of an effect on crossing scores than it does on crossing times.

### Crossing times prior to noise exposure

Baseline crossing times for noise exposed and control rats over the 19-week period were pooled and compared with a repeated measures ANOVA, followed by multiple pairwise comparisons of individual weeks. Overall, there is a highly significant effect of training week across the balance beam crossing time data [*F*_(18_,_4914)_ = 5.1, *p* < 0.00001]. This effect was investigated further with multiple pairwise comparisons. Most timepoint during the training period (Figure 3, yellow box) were greater than the timepoint during the proficient period (Figure 3, green box, ^). There is only one timepoint that is significantly slower than other training weeks (−16 weeks). During week 16 there were 27 trials that took longer than 3 s, including 6 failures due to crossing times that exceeded 10 s. It is likely that the high number of failures along with an otherwise higher mean drove up the mean for this week to an outlier level. We believe that variability in crossing is a normal part of early training and is a good indicator of need for additional training. There are also two timepoint (−11 and −9 weeks) that were significantly faster than other training timepoint. Crossing times below the cut-off for proficiency even though they are not yet stable suggests that rats are becoming more proficient in the balance beam crossing task near the end of the training period. No other training timepoint were significantly different from one another. Once we attained a proficient level of training in weeks −7 to −1 (Figure 3, green box), variability was low and there were no significant differences in crossing time between any timepoint.

Baseline crossing scores for noise exposed and control rats over the 19-week period were compared with a repeated measures ANOVA. Overall, there is a highly significant effect of training week across the balance beam crossing score data [*F*_(18_,_5004)_ = 4.0, *p* < 0.00001]. This effect was investigated further by analyzing individual weeks with multiple pairwise comparisons. Every training week is significantly different from at least one other training timepoint and at least one proficient timepoint except for week −8, which is only different from other training timepoint (weeks −19 to −12; Figure 4). Once rats become proficient, scores stabilize with little variability and no significant differences are observed between any proficient timepoint. Unlike the changes observed in crossing times after noise exposure, crossing scores do not increase to levels above the cut-off for proficiency in the weeks after noise exposure (Figure 4, red circles) even though they are greater than control crossing scores. This suggests that our scoring system is an effective tool for tracking training and determining when rats are ready to be noise exposed but may not be sensitive to noise-induced changes in motor performance.

### Increased balance beam crossing time is observed after noise

**FIGURE 5:**
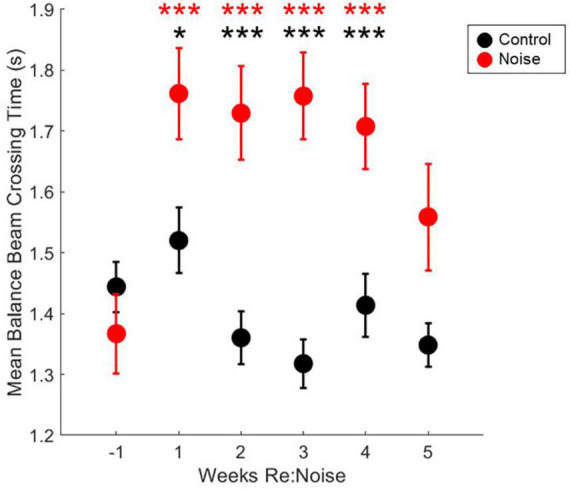
Crossing times are elevated after noise exposure. After noise exposure (red circles), mean balance beam crossing times are significantly elevated from baseline (red asterisks; *n* = 12; ***p < 0.001). Mean control balance beam crossing times (black circles) are not different from baseline at any timepoint (*n* = 8). At baseline, noise and control crossing times are not significantly different. However, during weeks 1–4, control and noise mean balance beam crossing times are significantly different (black asterisks; ***p < 0.001; *p < 0.05). Mean noise values are produced from a total of 240 individual data points per experimental week. Mean control values are produced from a total of 160 individual data points per experimental week. Mean values are plotted with standard error of the mean (SEM).

Six timepoint were compared [−1 (baseline), 1, 2, 3, 4, and 5-weeks post-noise] in 12 noise exposed and 8 control rats. A total of 10 observations per experimental day twice per week, per rat were analyzed. Weekly crossing times were compared with repeated measures ANOVA and there was a significant effect [*F*_(5_, _1975)_ = 5.2, *p* = 0.0001]. Differences within groups across experimental weeks and differences between control and noise exposed rats were analyzed with multiple pairwise comparisons. In controls, there were no significant differences between any timepoint (Figure 5). There was a significant difference between baseline (Mean = 1.37, SEM = 0.07) and post-noise data collected at 1-week (Mean = 1.76, SEM = 0.08, *p* < 0.0001), 2-weeks (Mean = 1.73, SEM = 0.08, *p* < 0.0001), 3-weeks (Mean = 1.76, SEM = 0.07, *p* < 0.0001), and 4-weeks after noise exposure (Mean = 1.71, SEM = 0.07, *p* < 0.0001), but not 5-weeks after noise exposure (Mean = 1.56, SEM = 0.09, *p* = 0.17). When control and noise exposed rats were compared to each other at each timepoint, control (Mean = 1.44, SEM = 0.04) and noise baselines were the same (*p* = 0.21); however, control and noise rats were different 1-week (Control Mean = 1.52, SEM = 0.05; *p* = 0.02), 2-weeks (Control Mean = 1.36, SEM = 0.04; *p* = 0.0002), 3-weeks (Control Mean = 1.32, SEM = 0.04; *p* < 0.00001), and 4-weeks (Control Mean = 1.41, SEM = 0.05; *p* = 0.003), but not 5-weeks after noise exposure (Control Mean = 1.34, SEM = 0.04; *p* = 0.07).

### No change in balance beam crossing scores

**FIGURE 6:**
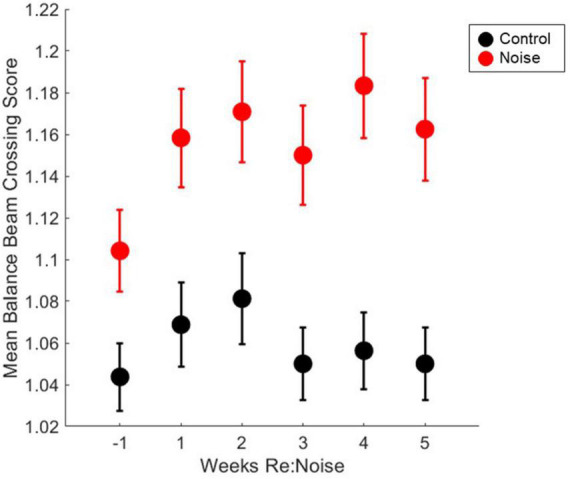
Crossing scores are unaffected by noise exposure. Control (black circles, *n* = 8) and noise-exposed (red circles, *n* = 12) mean balance beam crossing scores are not significantly elevated from baseline (*n* = 12). Mean noise values are produced from a total of 240 individual data points per experimental week. Mean control values are produced from a total of 160 individual data points per experimental week. Mean values are plotted with standard error of the mean (SEM).

Six timepoint were compared [−1 (baseline), 1, 2, 3, 4, and 5-weeks post-noise] in 12 noise exposed and 8 control rats. A total of 10 observations per experimental day twice per week, per rat were analyzed. Weekly crossing scores were compared with repeated measures ANOVA and there was not a significant effect [*F*_(5_, _1990)_ = 0.52, *p* = 0.7647]. Mean crossing scores remained stable for each group after initial training (Figure 6) and although scores appeared elevated following noise exposure multiple pairwise comparisons were not carried out due to lack of main effect.

In the week prior to noise exposure, there were no failures (scores = 3). After noise exposure, there was no significant change in the breakdown of the scores; however, there was a score of 3 assigned to one trial of the 240 collected from the group of 12 noise exposed rats during the two experimental days recorded during these weeks (Table 1). These were the only failed trials observed in this cohort of proficiently trained rats. Thus, the data suggest that after rats become proficient in the balance beam crossing task, noise exposure has little to no effect on their ability to walk smoothly across the balance beam without stopping.

**TABLE 1:** Rat crossing scores before and after noise exposure.

Score	Week −1	Week 1	Week 2	Week 3	Week 4	Week 5
1	203	203	199	206	197	204
2	37	37	41	33	43	35
3	0	0	0	1	0	1

Rat crossing scores are broken down 1 week before noise exposure and out to 5 weeks after noise exposure. Score of 1 = No stops, Score of 2 = 1–3 stops, and Score 3 of 3 = 3 + stops or > 10 s to complete the task.

### Number of stops per rat before and after noise exposure

**FIGURE 7:**
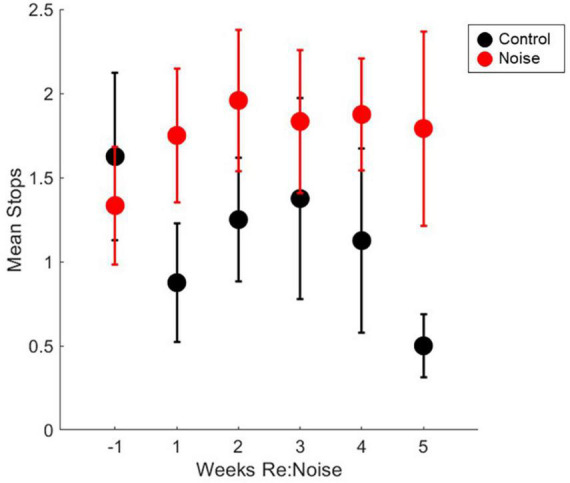
Number of stops during balance beam crosses are not significantly affected by noise exposure stops in control (black circles, *n* = 8) and noise-exposed (red circles, *n* = 12) groups are not significantly elevated from baseline (*n* = 12). Mean noise values are produced from a total of 240 individual data points per experimental week. Mean control values are produced from a total of 160 individual data points per experimental week. Mean values are plotted with standard error of the mean (SEM).

Stopping is a known issue in evaluation of motor performance with balance beam tasks. To address this issue, we used an additional assessment of stops to identify behavioral changes in addition to walking speed. Although our scoring method is based primarily on stops, it also takes failed trials into consideration when a score of 3 is assigned. This is not possible if stops are directly quantified. The fact that the scoring method, does not directly quantify stops could be a factor for the changes in crossing time since a score of 2 can be a trial with 1, 2, or 3 stops, and it is possible for the number of stops to increase without a change in score. To further investigate the impact of stopping during balance beam crossings, we took the scoring data one step further. By reviewing the number of stops that occurred each week in control and noise exposed rats, we can confirm that stopping is not a factor in the significant changes observed in balance beam crossing times. Stops that occurred during each experimental timepoint (2 trials per week) were totaled for each rat, and weekly group data were compared with repeated measures ANOVA. There was no effect of experimental week on stopping during balance beam crosses [Figure 7; *F*_(5_,_90)_ = 1.89; *p* = 0.1045]. Due to a lack of main effect, multiple pairwise comparisons were not performed. This finding further supports the idea that rats move more slowly across the beam after noise exposure and are not stopping a greater amount than non-noise exposed rats (baseline or control).

## Discussion

We have demonstrated a significant increase in balance beam crossing time in rats exposed to 120 dB SPL 3OBN. This amount of noise has been shown to abolish or reduce VsEP responses and has been linked to decreased calretinin-immunoreactivity of calyx-only afferent terminals in the saccule (Stewart et al., 2020). In human subjects, slower walking speed has been linked to blast-induced vestibular impairment (Akin et al., 2022), age-related imbalance (Xie et al., 2017), and increased risk for falls that can result in serious injury or loss of independence (Montero-Odasso et al., 2005). Although we can assume that our noise exposure paradigm induced similar damage as Stewart et al. (2020), in the current study, physiological and anatomical data are not available. Future studies will explore damage at the cellular and physiological level in relation to motor impairment to directly support a vestibular origin for this deficit.

**FIGURE 8:**
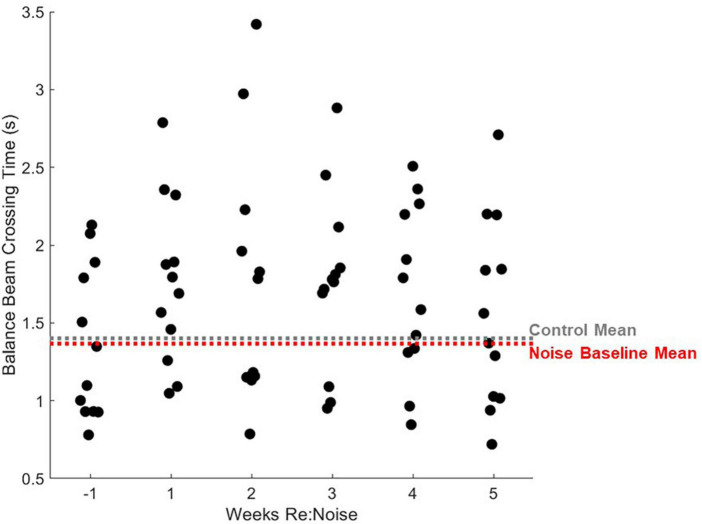
Individual rats’ balance beam crossing times. Mean balance beam crossing times averaged from 20 trials per week are plotted for each rat that was noise exposed (*n* = 12). For comparison, mean control balance beam crossing times for weeks −1 to 5 are plotted as a horizontal gray dashed line and mean baseline (week −1) times are plotted for noise-exposed rats. Mean non-noise exposed rats are extremely similar to control rats. After noise exposure, most noise exposed rats’ mean crossing times are greater than the mean. The highest balance beam crossing time is seen 2 weeks after noise exposure. After this, balance beam crossing times show some improvement and looks more similar to baseline in most rats by week 5.

**FIGURE 9:**
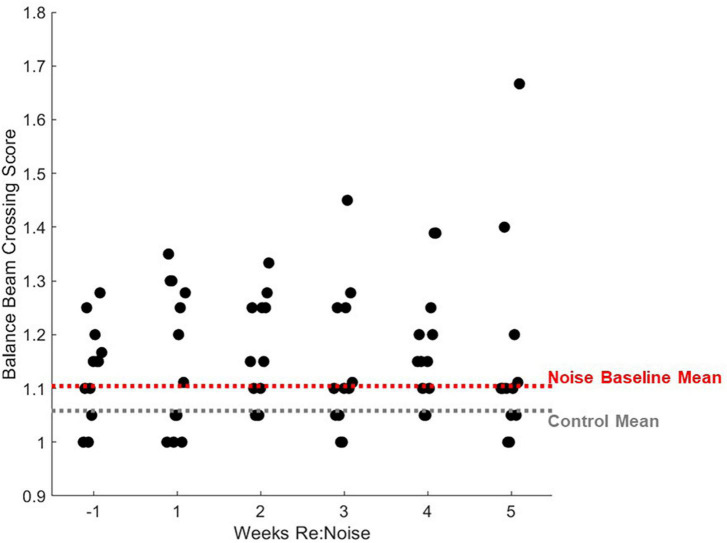
Individual rats’ balance beam crossing scores. Mean balance beam crossing scores averaged from 20 trials per week are plotted for each rat that was noise exposed (*n* = 12). For comparison, mean control balance beam crossing scores for weeks −1 to 5 are plotted as a horizontal gray dashed line and mean baseline (week −1) scores are plotted for noise-exposed rats. Mean non-noise exposed and baseline noise-exposed rats are similar. After noise exposure, the distribution of crossing scores does not change significantly with the exception of one outlier data point that is related to increased stopping (Figure 10).

**FIGURE 10:**
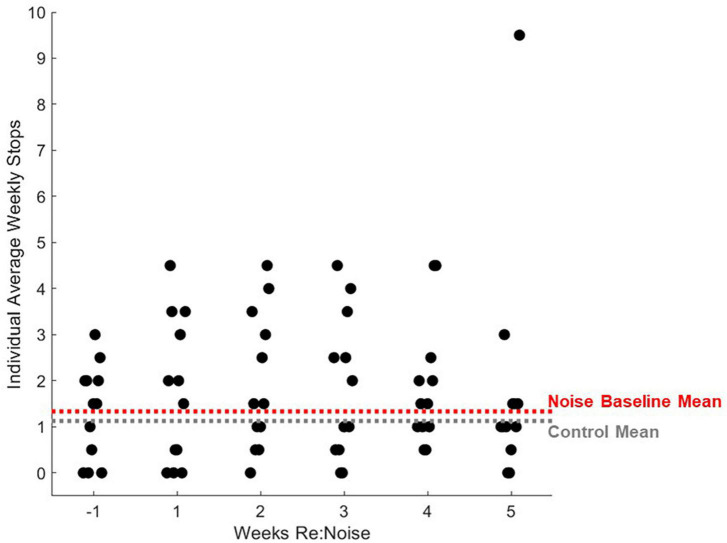
Individual rats’ balance beam crossing stops. Stops during balance beam crossing were totaled for each experimental day and 2 days per experimental week were averaged for each noise exposed rat (*n* = 12). For comparison, mean control stops during balance beam crossing for weeks −1 to 5 are plotted as a horizontal gray dashed line and mean baseline (week −1) stops are plotted for noise-exposed rats. Mean non-noise exposed and baseline noise-exposed rats are similar. After noise exposure, the distribution of stops during balance beam crossing do not change significantly with the exception of one outlier data point.

Interestingly, a small number of rats were relatively insensitive to noise exposure. Figure 8 shows data for each noise exposed rat plotted by week (black circles). The average of all control data from weeks −1 to 5 (gray dashed line) and the mean baseline data for the noise group (red dashed line) are nearly identical. The week after noise exposure, crossing times for most rats were above the mean. However, at least 3 rats exhibited little or no increase in crossing time despite their having been noise exposed. Indeed the individual data illustrates a highly variable effect of noise on crossing time suggesting that some rats were relatively insensitive to noise exposure. Individual rats’ balance beam crossing scores (Figure 9) and the number of stops observed in the weeks following noise exposure (Figure 10) were unaffected by noise exposure versus baseline with the exception of one outlier rat that stopped more, leading to a higher score in week 5. This may reflect physiological difference in noise sensitivity or, possibly, differences in behavior during noise exposure that allowed some rats to reduce their actual exposure (e.g., finding a quieter corner in the exposure box or positioning their head so as to protect at least one ear from the noise). An additional caveat is that we were unable to track estrous cycles in this female cohort of rats. It is possible that noise exposure had a varying impact on rats due to the phase of their estrous cycle, and it is known that estradiol protects against noise-induced hearing loss in mice (Shuster et al., 2021). Because we see varying levels of damage across individual rats’ crossing times (Figure 8) with some rats showing severe impairment and others seemingly unaffected, it is possible that higher levels of estradiol in some rats due to estrous cycle phase may have played a protective role against noise-induced impairment. Future work should consider protective effects of estrogen since there may be a link between this hormone and vestibular disorders (for review, Khiati et al., 2022). We note, however, that an earlier preliminary study of male rats’ crossing times in a similar task produced similar findings; some animals exhibited slower crossing times but others were unaffected (Stewart, unpublished data).

Noise exposure has been reported to impair balance beam crossing previously in young mice (Tamura et al., 2012). In that study, 70 dB SPL high-frequency (16 kHz) or low frequency noise (100 Hz) was delivered for 1 month. Mice from control, high frequency and low frequency groups were compared, and only low frequency noise impaired vestibular structure and function. Low frequency noise (LFN) caused hair cell loss and oxidative stress. It also caused impaired balance beam crossing, poorer rotarod performance, and changes in gait.

### Genetic models

Multiple studies in mice demonstrate that balance beam crossing times are sensitive to vestibular abnormalities produced by genetic manipulations affecting vestibular related structures. For example, multiple published studies report that knock-out mouse strains with vestibular abnormalities exhibit slower crossing times on balance beam tasks. The P2X_2_ receptor gene is associated with supporting cells and transitional cells in vestibular sensory epithelia. In P2X_2_ knock-out mice, Takimoto et al. (2018) reported a significant increase in balance beam crossing speed on a smaller (10 mm), but not larger beam (20 mm). Knock-out mice also exhibited decreased VOR gains but no change in optokinetic reflex (OKN) or locomotor activity suggesting a vestibular-specific deficit related to the P2X_2_ gene (Takimoto et al., 2018). Manes et al. (2019) showed that a mouse strain lacking otopetrin 1 lacked otoconia and exhibited reduced locomotion and rearing frequency, increased grooming, and significantly reduced scores on a balance beam task. Finally, FGF12 knockout mice were slower to cross a balance beam, slipped more, and fell sooner off a rotarod (Hanada et al., 2018).

### Chemical exposure

Sen et al. (2020) report that mice fed 0.2% cuprizone for 37 days showed no change in nociception or rotarod performance but walked more slowly on ladder and balance beam tasks. Cuprizone is a compound commonly used to study demyelination and gliosis in the central nervous system. Balance beam crossing was slower 32–35 days after starting the diet. Recovery after discontinuation of the diet was not assessed. These mice had no gross motor abnormalities, but changes were observed in tissue from cortex, cerebellum, and brainstem, including oligodendrocyte loss and gliosis in the vestibular nuclei.

Negishi-Oshino et al. (2019) exposed mice to iminodipropionitrile (IDPN), a nitrile-related chemical known to dose-dependently impact vestibular hair cells (Llorens and Rodríguez-Farré, 1997; Soler-Martín et al., 2007). They reported shorter rotarod times and increased balance beam crossing times, as well as slower air-righting reflexes. IDPN exposed mice had significant hair cell loss in saccule, utricle, and cristae. Reduced cVEMP amplitudes and prolonged latencies were observed consistent with the reported hair cell losses. Times to traverse the balance beam and rotarod fall times were correlated with abnormal cVEMP findings.

### Lesions

In one of the few studies that used rats (Modianos and Pfaff, 1976), lesions placed in the lateral and superior vestibular nucleus and the inferior olive were related to failed balance beam crossing performance. Lesions of the medial medullary reticular formation and medial vestibular nucleus had less effect and rats with lesions of the cerebellar nuclei were unimpaired on the balance beam task. Heskin-Sweezie et al. (2010) reported balance beam crossing deficits in mice 4 and 19 h after unilateral vestibular deafferentation, and compensation within 44 h post-lesion.

Similar to these studies, we found significant changes in balance beam crossing times in rats exposed to noise that had been previously demonstrated to attenuate VsEP responses and reduce calretinin immunolabeling of calyx-only afferents in the sacculus (Stewart et al., 2020). Importantly, our report is one of the first to document extended training and recovery of walking speed following noise exposure. Although we focused on short-term effects of noise exposure and recovery (5 weeks); future studies are needed to evaluate the effects of multiple noise exposures and potential longer-term effects of noise exposure.

It has also been shown that mice with compromised striolar zones caused by a genetic knock out move more slowly when traversing a balance beam (Ono et al., 2020). Although our noise exposure model produces a lesser deficit than the knockout described by Ono et al. (2020), we were able to demonstrate noise-induced changes in striolar zone calyx-only labeling with attenuated VsEP responses previously with the same noise exposure model used in this study (Stewart et al., 2020). Future studies should evaluate noise exposures that do not produce permanent VsEP deficits, such as the 110 dB noise exposure model described by Stewart et al. (2021) to determine if there are cumulative effects of multiple exposures that individually exhibit recovery. If such exposures were to accelerate age-related vestibular loss, similar to age-related hearing loss described by Altschuler et al. (2022), that would have significant implications for the general population and would imply that cumulative environmental noise exposure is correlated with changes in vestibular impairment and concurrent motor function. It is also possible that an interaction between noise exposure and age may produce a greater effect than Tung et al. (2016) observed with age alone. This issue could be addressed in future studies that examine balance beam crossing performance in older rats with noise exposure history.

### Training may be important for establishing consistent balance beam crossing behaviors

Based on the pre-noise data (Figures 3, 4), we do not believe that prolonged training had an adverse impact on walking performance. In fact, prolonged training likely reduced variability and allowed us to identify effects specific to noise rather than changes related to novelty or fear. Despite a previous report that cautions against over-training as it can lead to mice stopping more during the tasks, turning around, moving the opposite direction, and overall miscalculated motor capabilities in mice (Tung et al., 2014), our study demonstrates the value of training to attain consistent balance beam crossing behavior. Particularly in early training, large changes and variability in performance in the absence of any treatment or experimental manipulation may occur. If one tracks balance beam training with a scoring system such as the one described in this study, outcomes are likely to have greater power due to rigorous training and well-documented baselines. It is possible that our work, in contrast to Tung et al. (2014), highlights an important difference between rat and mouse balance beam training, as other mouse studies have also used minimal training (Sen et al., 2020; Tkac et al., 2021). In rats, some studies provide training (e.g., Kucinski et al., 2013, 45 days), some see no effect of prior behavioral experience (e.g., Wallace et al., 1980), and others only provide brief training (e.g., Drucker-Colín and García-Hernández, 1991). Although this work featured a prolonged training period of 19 weeks, it might not be necessary to train for such an extended period of time in future studies. Figure 4 shows that once scores reach a proficient level (<1.2), they become stable below the cut-off for proficiency (<1.2) regardless of noise exposure. Noise did not produce dramatic increases in scored behavior vs. pre-noise baseline (Figure 6) which implies our scoring system is somewhat insensitive to noise-induced vestibular loss. This may suggest that although scores do improve with training, they are not an ideal tool for comparing the effects of various treatments in different groups of rats. Crossing time was extremely similar between noise-exposed and control rats at baseline, making it an appealing metric for assessment of treatment effects, in this case, effects of noise exposure. We believe that balance beam crossing time is a valuable behavioral metric signaling changes in vestibular-mediated motor performance due to peripheral noise-induced vestibular loss (Figure 5).

Taken together, these data confirm prior studies of vestibular-mediated motor impairment and demonstrate an effect of noise-induced vestibular loss in this impairment. The noise exposure paradigm used in this study has been described by Stewart et al. (2018, 2020) previously. It was known that this noise exposure paradigm abolished VsEP responses to small stimuli (Stewart et al., 2018, 2020) and attenuated responses to larger stimuli (Stewart et al., 2020). Decreased amplitude and prolonged latency in responses that could be measured, and a clear loss of some responses entirely, suggesting a threshold shift linked to reduced calretinin immunoreactivity of calyx-only afferent terminals (Stewart et al., 2018, 2020). We now report that noise exposure that causes cellular and electro-physiological changes in the vestibular periphery is also associated with slower walking speed in highly trained rats. This finding is particularly relevant to aging adults with noise exposure history, as their history may be linked to earlier changes in walking speed linked to imbalance and fall risk. This study also suggests that cVEMP testing may be a useful tool to identify vestibular-specific motor impairment vs. general age-related changes in peripheral musculature or neurological function in older adults. Slower walking speeds in conjunction with cVEMP findings could be used to determine the best course of treatment to reduce fall risk. This study did not use other established metrics for assessing vestibular dysfunction in rodents. Although batteries of motor tests that include rotarod, locomotor gait analysis, swimming, or vestibular reflex measurements might increase clinical relevance, our results are robust and reflect a significant change in motor performance after a single noise exposure. Other tests reduce the need for extended training and would provide confirmation as well as additional information about the nature of observed changes. Future work will examine repeated lower-level noise exposure paradigms that are more relevant to the human experience, and will examine the interaction between noise exposure and aging in the vestibular periphery.

## Data availability statement

The raw data supporting the conclusions of this article will be made available by the authors, without undue reservation.

## Ethics statement

The animal study was reviewed and approved by the Institutional Animal Care and Use Committee, VA Ann Arbor Healthcare System.

## Author contributions

CS, DB, DSB, RA, and WK conceived, designed the research, and interpreted results of the experiments. CS, DB, and MH performed the experiments, analyzed the data, and prepared the figures. CS and DB drafted the manuscript. CS, DB, MH, RA, and WK edited and revised the manuscript. CS, DB, DSB, MH, RA, and WK approved final version of the manuscript. All authors contributed to the article and approved the submitted version.
